# Fabricating ultra-thin nanofiber structures towards the advanced MEA of fuel cells: investigation of the degree of alignment, diameter, bead generation, and precision with Taguchi designs

**DOI:** 10.1039/d4na00558a

**Published:** 2024-09-30

**Authors:** Muhammad Yusro, Viktor Hacker

**Affiliations:** a Institute of Chemical Engineering and Environmental Technology TU Graz 8010 Austria yusro@student.tugraz.at; b Telkom University Jalan D.I. Panjaitan 128 Purwokerto 5317 Jawa Tengah Indonesia

## Abstract

Modified nanofibers with a more aligned and very thin structure are a potential approach owing to their promising properties and enhanced fuel cell performance compared to conventional randomly oriented nanofibers. The strategy to fabricate randomly oriented nanofibers has been widely explored. However, the key factors of oriented fibres, such as the degree of alignment, diameter, bead generation, and precision, have not been investigated in detail. In this work, four specific profiles of nanofibres related to the parameters of the electrospinning system were analysed in more detail for the first time: collector speed, distance, voltage, and nozzle movement. The concentration was set at a constant value of 10% (w/w) of polyvinyl alcohol (PVA), which is suitable for the potential application of fabricating membrane electrode assemblies (MEAs) for fuel cells. The results indicated that the applied electrical voltage is the most important factor among all the features. Taguchi methods implemented in this work revealed the correlation factors for each specific parameter.

## Abstract 2

Factors influencing the production of nanofibre MEA, such as the degree of alignment, diameter, beading and precision of the electrospun nanofibres.

## Graphical abstract

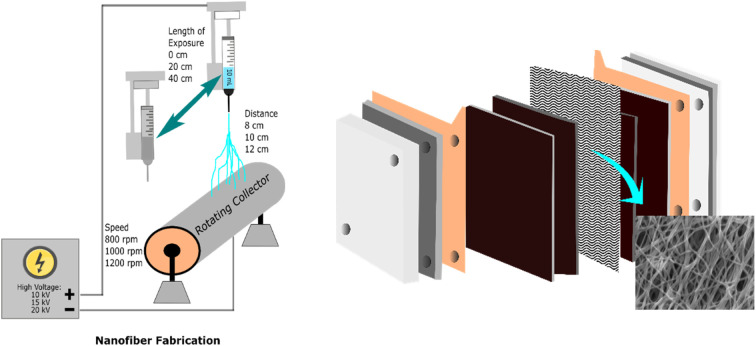

## 1. Introduction

**Fig. 1:**
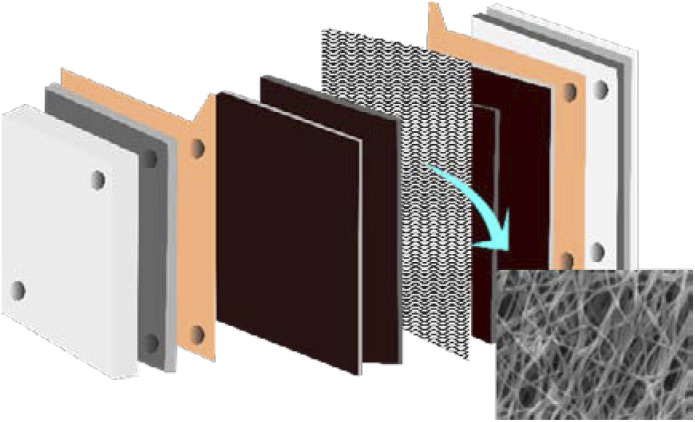
Implementation of nanofibers as MEAs in fuel cell applications.

Looking at the heart of a fuel cell, the membrane electrode assembly (MEA) is the part that is responsible for converting chemical energy into electrical energy *via* the electrochemical reaction separating electrons. Arranged at the center of the fuel cell architecture (illustrated in Fig. 1) is a membrane, which is the space that allows the movement of ion charges across different electrodes (cathode or anode), and electrode layers, which allow catalyst materials such as Pt to support important steps in electrochemical processes, for example, oxygen reduction reactions (ORRs) or hydrogen evolution reactions (HERs).^1^ This component requires superior mass transport, high ionic conductivity, mechanical integrity, and chemical stability.

Nanofibers, which are advanced materials, have widely been reported to enhance the specific characteristics of a material owing to their higher surface area and porosity. These advantages are essential to explore the strategy in the strategic fields, including fuel cell which need to enhance specific properties by using the materials to improve their technology performance. Nanofibers are popularly fabricated through electrospinning, considering this approach mixes several materials to obtain desired properties. Moreover, electrospinning works on a laboratory scale for research and development and could be easily scaled up to the industrial level.

The fabrication of nanofibers using electrospinning is challenging considering that this method employs electrostatic forces, which can be influenced by complex factors. The influencing factors in electrospinning can be classified into three categories: fluid, set-up, and ambient factors. These three influencing factors are orchestrated in the fabrication process using electrospinning to obtain the desired product. If the parameters do not fit enough to create a fibre, the result of electrospinning can be beads or even droplets. A detailed discussion on the influencing factors of electrospinning that affect nanofiber and bead formation can be found in previous studies.^2,3^

Modified nanofibers have gained attention because of their promising results, which make their structure superior to conventional ones. Theoretical explanation and experimental observation encourage that one of the modified nanofibers, which are aligned and thin, is recommended for the next level development of nanofiber fabrication through the electrospinning method to enhance the properties of manufactured products.

Ultra-thin nanofibers have been widely reported and implemented in various applications. By modifying the fiber into a thinner dimension, the surface area and the porosity are increased, which leads to enhance the characteristics of the desired products such as membranes or catalysts. In wood pulp invention, it has been stated that the a highly porous nanofibrous support owing to a thinner fibre dimension provided an expectedly higher flux than that of conventional membranes.^4^ In the fuel cell itself, an ultrathin silica layer has been fabricated to establish a highly durable carbon nanofiber applied as a carbon support for polymer electrolyte fuel cells. It has been mentioned that this ultrathin structure endows great potential to reduce the presence of water molecules in the neighborhood of the carbon supports and the OH^−^ radicals formed on the surface of the Pt catalyst.^5^

Regarding the orientation of the fibre, from a theoretical mechanical property point of view, changing the direction of orientation leads to better results bearing force load than the random structure. Experimentally, it has been reported that the oriented uni-axial formation could enhance up to 600% of modulus using poly(ε-caprolactone) (PCL) for tissue engineering.^6^ The simulation of the aligned nanofiber has also been conducted by modeling resistors with a conductivity analogous to the fibre conductivity.^7^ The aligned nanofiber with a more oriented axial found has superior conductivity due to less obstacle to moving to the other side. A large body of evidence has also proved that the aligned nanofiber increases the anionic conductivity 10–15 times, promising to enhance the fuel cell performance.^8^

The strategy to fabricate a modified nanofiber has been resumed and elaborated in a previous publication.^9^ The key to modulate the nanofiber dimension is adjusting the influencing parameters of electrospinning, which are orchestrated during the fabrication process. Furthermore, the strategy to align nanofibers is conducted by managing fibre deposition when it sits in the collector. Nanofiber electrospinning conventionally forms by stretching and elongation due to the electrostatic motion force; the fibre spins and creates random formation when it reaches the collector (caused by electricity and then spin = electrospinning). The strategy to manage this spinnability process by controlling the motion is relatively tricky. Different materials can cause different results influenced by sub-parameters of fluid factors such as conductivity or surface tension.

In this work, the Taguchi method was implemented as the design of experiment (DoE) to reduce the cost and time without downgrading the quality of research. The DoE has advantages correlating the process variables to assess the relationship between the variables and reducing the cost due to laboratory work or massive computer simulation.^10^ The Taguchi design is able to evaluate systematically the effect of factors.^11^ By this approach, the optimum conditions could be determined to improve the quality of the product, which are the degree of alignment, diameter, bead generation, and precision. This method could reduce the experiment by implementing a pair of combinations rather than having to examine every possible combination.^12^ This method has also reportedly been highly implemented to optimize and analyze the relationship between electrospinning parameters and the nanofiber diameter.^13–23^

Even though the optimizing process of nanofiber has been reported in previous research, a more detailed and holistic investigation regarding its parameters and analytical assessment concerning how they influence the degree of alignment, diameter, beads generation, and precision have not been conducted yet. This work reported the first investigation regarding the influencing parameters of aligned nanofibers, including the speed of rotation, voltage, distance, and exposure area. Increasing rotation speed is chosen due to the simplest method to fabricate the aligned nanofiber. Voltage and distance are selected because both have the effect of stretching and elongating nanofibers. It has also been observed that the alignment of nanofibers is affected by modulating those factors in ceramic materials.^24^ Those parameters are considered set-up parameters that must be optimized and fixed for the versatile manufactured product. Moreover, the last factor, the nozzle movement correlated with exposure area, was decided to be assessed because when the nozzle is changing direction, the stability of the structure of nanofiber can be changed. It should be noted that changing the concentration of the catalyst could fluctuate the fuel cell performance.^25^ The concentration, which is reported as the most influencing factor in electrospinning, is set at a constant value.

## 2. Experimental

### 2.1. Preparation of the solution

PVA (Sigma Aldrich USA, 98–99% hydrolyzed, MW 31.000–50.000) was used in this experiment. PVA is a well-known material because it has high electrospinnability, and this polymer has been used in fuel cells for the membrane.^26,27^ Since most references used this value concentration, 10% (w/w) was chosen as the permanent concentration.^2,3,28–30^ PVA powder was dissolved in distilled water using a magnetic stirrer for 2 hours at 85 °C temperature until the solution became homogenous. The PVA solution was rested for 24 hours to cool down and support disappearing bubbles.

### 2.2. Electrospinning apparatus

A Tong Li-Tech electrospinning machine (Model No. TL-Pro, Tong Li Tech Co., Ltd, China) was used to fabricate the nanofiber. This electrospinning machine has a rotating collector; the ratio speed can be adjusted. Furthermore, the area of exposure can also be managed. The parameters of electrospinning that were not varied in this experiment include a fluid flow of 0.01 ml per hour and the employment of the spinneret tip number 23. The temperature was set at room temperature.

### 2.3. Measurements

The nanofiber was investigated simply based on image data acquired by Scanning Electron Microscopy (SEM). Instead of using the Fast Fourier Transform, which investigates the whole image by converting to a grey scale.^31,32^ The formula measures the degree of alignment for each fibre. This approach follows eqn (1):

1 DoA = |cos *θ*| × 100%

where *θ* is the angle of the assessed individual fibre between the fibre direction and the desired direction. The desired direction refers to the orientation, which is pointed out as the baseline axis that has 0° or 180° (both degrees have a maximum cosine value of 1). The other 99 fibres would be compared to that axis. The DoA values vary from 0 (random orientation) to 1 for perfect alignment formation.^33^ By multiplying the value by 100%, the maximum DoA will be 100%.

This formula is simpler than that reported previously.^33,34^ The average of all the fibre's diameter and their standard deviation (STD) was calculated based on 100 individual fibres. Furthermore, the standard deviation was calculated with precision. Bead generation was manually calculated based on their appearance at 10 000× magnification for all the experimental scenarios. In this experiment, an image processing program was conducted using ImageJ. Meanwhile, statistical measurement and visualization were performed using Microsoft Excel and also Origin.

### 2.4. Taguchi design: method and analysis

The Taguchi method is straightforward, using an orthogonal array to decide the number of experiments. This method implements an arranged orthogonal array that needs to be organized and involves factors that affect the quality of the product.

In the Taguchi design, determining the parameter design is the most critical step in achieving quality while reducing the cost.^35^ In this experimental design, the level of the factors, which are the degree of alignment, diameter, bead generation, and precision of nanofibers, needs to be varied to seek the trends of responses. These responses will be assessed based on the four factors (rotation speed, voltage, distance, and exposure) with three-level values based on hypothetical values: high, intermediate, and low. The orthogonal formula L9(3^4^) is applied, resulting in nine experiments (27 in total with three replications) with variables. This experiment design reduced cost and time compared to the complete factorial design, which is 3^4^ = 81 experiments.

The step-by-step instructions to decide on the parameter design of the Taguchi method are as follows:

(1) Defining the quality characteristic is the attribute that should be optimized.

(2) Deciding the number of levels for the design factors.

(3) Designing the experiment's matrix and determining the data analysis technique.

(4) Conducting research based on the matrix of the experiment.

(5) Investigating the experimental results based on the S/N ratio and ANOVA studies.

(6) Selecting the optimal values of parameters and predicting the desired performance; and

(7) Verifying the optimal design parameters by the confirmation experiment.^36^

The experimental parameters and their levels are given in Table 1. The values for levels 1 to 3 are chosen considering that a higher-level value of each factor hypothetically will get the better result or, in this case, a better-aligned nanofiber direction.

**Table 1:** Selected factors of electrospinning and their levels for Taguchi designs

No	Factors	Abbreviation	Units	Level 1	Level 2	Level 3
1	Speed	S	rpm	800	1000	1200
2	Voltage	V	kV	20	15	10
3	Distance	D	cm	12	10	8
4	Movement	M	cm	40	20	0

In this method, the signal-to-noise (S/N) ratio needs to be determined to investigate each factor's influence on the final responses and specify the optimal level for each parameter. The loss function of the Taguchi method was transformed to a signal-to-noise (S/N) factor to determine the most significant levels of control factors; in this method, the higher S/N ratio refers to the optimum level of the control factor.

Considering that the higher value of the degree of alignment represents better results, the-higher-the-better was chosen to define the S/N ratio following eqn (2):^37^2
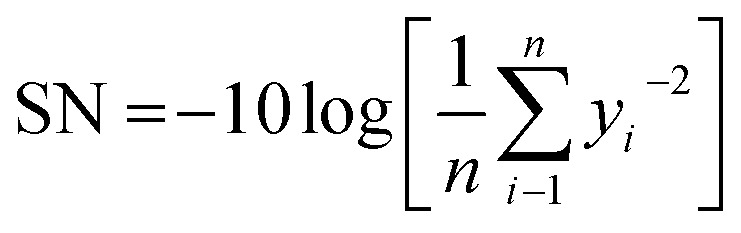


Meanwhile, the diameter, bead generation, and precision are better at lower values, so that these three responses follow the-lower-the-better, which is represent by eqn (3):^10^3
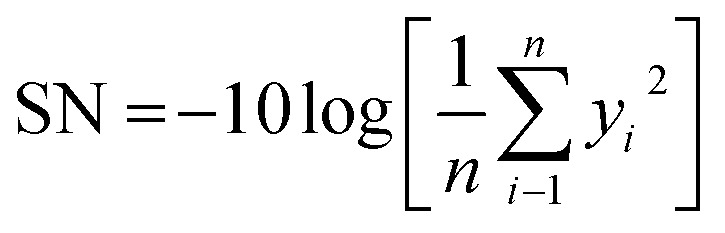


**Fig. 2:**
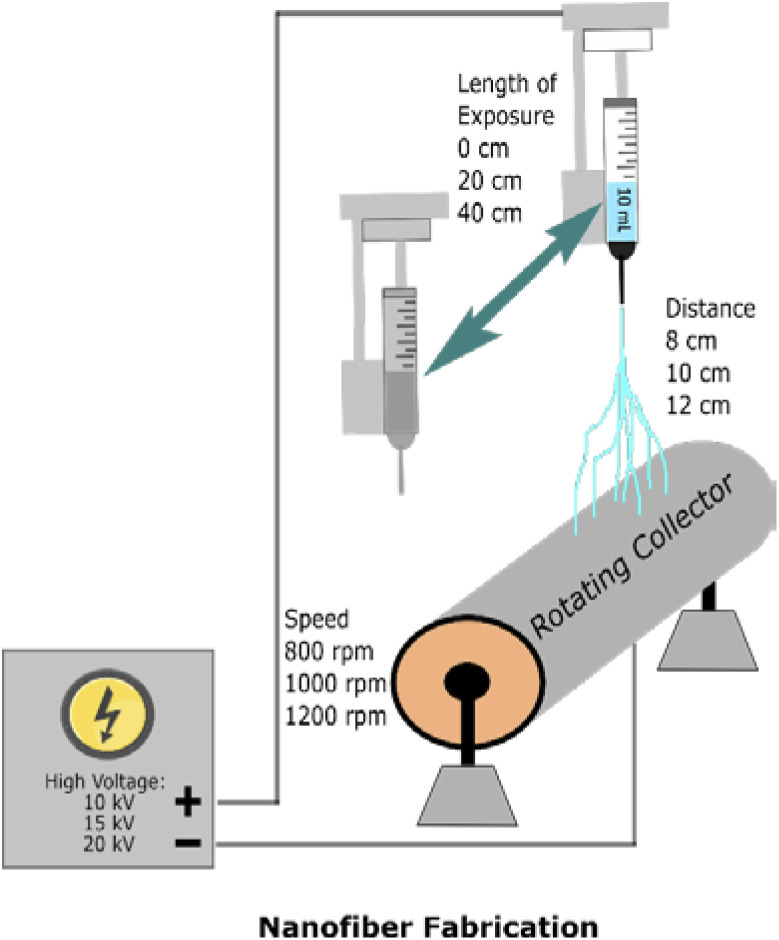
Illustration of the design of experiment for nanofiber fabrication.

In these equations, SN is defined as the signal-to-noise ratio (dB), *y*_*i*_ is the experimental response, and *n* is the number of experiments. All the design experiments conducted in this study are summarized in Fig. 2.

## 3. Results and discussion

### 3.1. Nanofiber structure assessments

Based on the investigation using the ImageJ software, the measurement of the degree of alignment, diameter, bead generation, and precision for the nanofiber was carried out. The results of these four parameters are presented in Table 2.

**Table 2:** Results of the Taguchi design and Taylor cone observation

Exp. no.	Factors				Measurement (100 fibres)				Taylor cone
	*S* (rpm)	*V* (kV)	*D* (cm)	*M* (cm)	DoA (%)	Diameter (nm)	Bead	Precision	
1	800	20	12	40	90.37	27.62	3	12.23	Stable continues
2	800	15	10	20	86.26	29.8	20	13.93	Stable discrete
3	800	10	8	0	72.01	18.63	110	7.78	Stable continues
4	1000	20	10	0	83.26	22.49	47	10.7	Unstable discrete
5	1000	15	8	40	88.3	27.03	9	9.77	Unstable discrete
6	1000	10	12	20	82.31	17.67	82	7.22	Unstable droplet
7	1200	20	8	20	84.13	24.94	17	9.65	Unstable discrete
8	1200	15	12	0	91.03	18.68	56	8.95	Stable discrete
9	1200	10	10	40	81.63	19.03	92	7.43	Stable continues

**Fig. 3:**
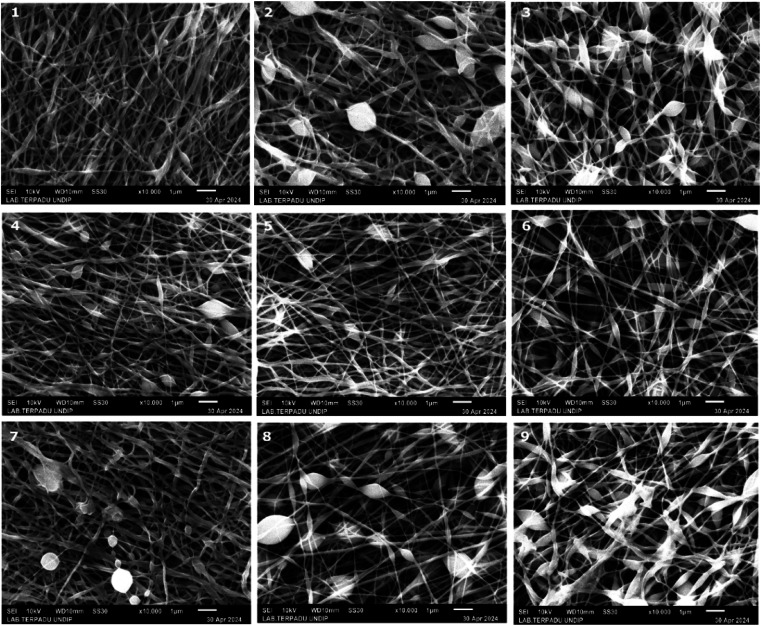
Image of the nine experiments with the Taguchi design captured *via* SEM.

**Fig. 4:**
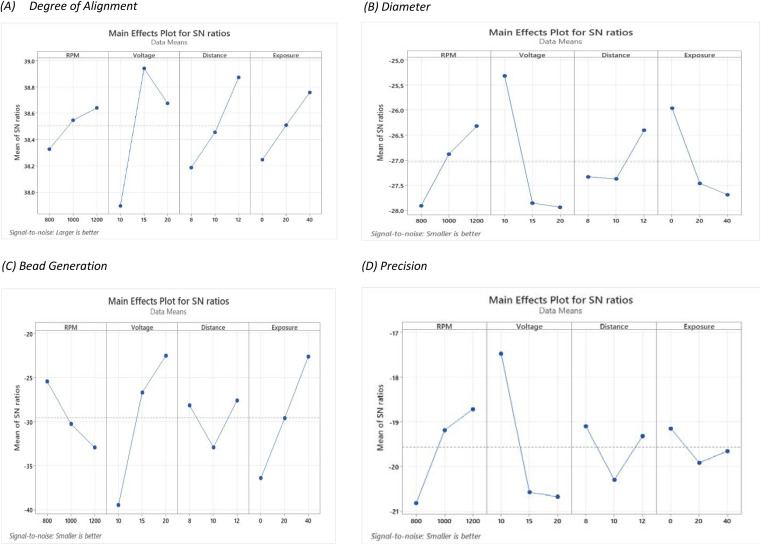
The main effect plot for SN ratios for each response correlated with each factor, including (A) degree of alignment, (B) diameter, (C) bead generation, and (D) precision.

In this table, the profile of Taylor cone has also been explored considering that the profile of Taylor cone influences the result of fibre structure for further analyses. To provide the actual image of nanofiber structure, Fig. 3 presents the image of each experiment of Taguchi design. It could be seen that the various structures of nanofibre correlate with the different parameter settings of electrospinning, which are modulated by Taguchi's design. Regarding the Taguchi design, the Main Effects Plot for the Signal-to-Noise (S/N) Ratio was constructed using the Minitab software. The effect of parameter for the desired factor is shown in Fig. 4.

#### 3.1.1. Degree of alignment

The degree of alignment is one of the key structures to be achieved, considering that this characteristic could boost the mechanical properties, ionic conductivity, and mass transfer.^9^ The degree of alignment means how close the fabricated fibre is to the desired orientation. To investigate the degree of alignment in nanofibers, 100 fibres were assessed using the ImageJ software. The selected fibre was measured by tipping a point at the measured fibre and then comparing it with the angle from the desired fibre axis. For instance, the desired fibre orientation is the horizontal axis. Therefore, if the measured fibres are also horizontal, the degree of alignment is 180. The value of Cosine 180 is −1, so that the absolute value is implemented in the equation. The cosine value transforms in percentage, making the highest degree of alignment 100%. However, the lowest degree of alignment occurs when the cosine value is 90 or 270. Under this condition, the measured nanofiber is perpendicular to the orientation of the desired nanofiber.

Based on Table 2, it could be seen that the lowest DoA, 72.01%, came from experiment no. 3. (*S*: 800, *V*: 10, *D*: 8, *M*: 0). However, the highest degree of alignment, which is 91.03%, was provided by experiment no. 8 (*S*: 1200, *V*: 15, *D*: 12, *M*: 0). Meanwhile, based on the S/N ratio (Fig. 4(A)), it could be concluded that the most aligned nanofiber will be achieved at *S*: 1200, *V*: 15, *D*: 12, *M*: 40.

Based on the result, it could be seen that even though theoretically higher speeds tend to a higher orientation of nanofiber. In this scale, the ultrathin nanofiber is mostly influenced by applied voltage. Based on the S/N ratio graph (Fig. 4(A)), the distance between the maximum and minimum values of S/N ratio in the voltage factor is higher than the speed of collector factors. This also indicates that applied voltage factors were dominant in this experimental design. The second factor to be concerned is also the distance between the nozzle and the collector; proper distance needs to be achieved to provide enough space for the fibre to be stretched and elongated in a more oriented direction.

#### 3.1.2. Diameter

Diameter is an important outcome that researchers assess when fabricating nanofibers using electrospinning. In the context of fabricating MEA, the dimension of nanofibers could be an interesting parameter to be investigated. Thinner nanofiber will provide a higher surface area and porosity. These structures have the potential to improve fuel cell performance. To investigate the nanofiber diameter, 100 fibres were also calculated using the ImageJ software. The scale bar from SEM processing was used as a reference to compare with the measured nanofiber. From this process, the size of the nanofiber and its standard precision were calculated.

**Fig. 5:**
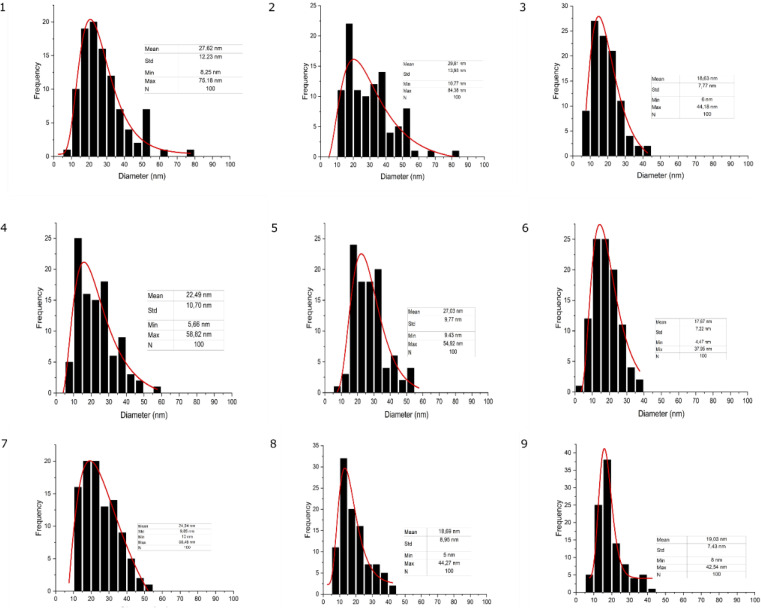
Measurement of the diameter of nanofibers (mean, standard deviation, minimum, and maximum size are provided).

Fig. 5 shows the distribution of fibres for each experiment. The mean, standard precision, minimum, and maximum fibre size are provided. Based on Table 2, it was determined that the smallest nanofiber produced by experiment no. 6 achieved 17.67 nanometers (*S*: 1000, *V*: 10, *D*: 12, *M*: 20). However, the biggest nanofiber dimension produced from experiment no 2 attained 29.8 nanometers (*S*: 800, *V*: 15, *D*: 10, *M*: 20). This nanofiber is thinner than the existing MEA, which is generally around 30–500 nm.^38–40^ Meanwhile, based on the S/N ratio (Fig. 4B), it can be concluded that the thinnest nanofiber will be achieved at *S*: 1200, *V*: 10, *D*: 12, *M*: 0.

Based on the S/N ratio comparison, it could be concluded that the applied voltage is the most significant factor to modify the nanofiber dimension. In the ultrathin scale, the result reveals that the lower voltage leads to a smaller nanofiber. This result is discrepant compared to the majority of the reported results, which state that increasing voltage leads to a decrease in fiber size.^19,21,41–43^ In this experiment, when a higher voltage occurred in the system, the electrical force strongly pulls the polymer to the collector. This event reduces the stretching action, making the polymer deposits a bigger nanofiber dimension.

#### 3.1.3. Bead generation

The fabrication of nanofibers using electrospinning is a challenge due to the undesirable structures, such as bead formation. Bead formation is a structure similar to a rugby ball with an elliptical form. This profile is caused by adjustment due to fibres in the fabrication process, which minimizes free energy in the system. This fine-tuning is done by reducing the surface area per mass unit, so that it forms ball.^2^ The bead structure caused non-uniform nanofiber features to disrupt the smoothness of the nanofiber. In the context of MEA fabrication, bead generation is avoided due to the non-uniformity of the structure.

In this work, bead generation was calculated manually from an image captured by SEM. Based on Table 2, less bead generation was achieved by experiment no. 1 with only 3 beads (*S*: 800, *V*: 20, *D*: 12, *M*: 40). Furthermore, the most bead formation appeared in experiment no. 3 with 110 beads (*S*: 800, *V*: 10, *D*: 8, *M*: 0). Meanwhile, based on the S/N ratio (Fig. 4(C)), it could be concluded that the most aligned nanofiber will be achieved at *S*: 800, *V*: 20, *D*: 12, *M*: 40.

Based on the S/N result, it could be seen that in this design, the experiment is strongly correlated with bead generation. The magnitude of S/N ratio is distinguished compared to the other responses such as diameter and precision. In bead formation, the applied voltage becomes the most significant factor. It should be noted that bead formation is also correlated with the diameter fibre; if the bead is generated, the size of the nanofiber also decreases.^3^ It could be seen that the profile of S/N ratios in bead formation is reversed to diameter.

#### 3.1.4. Precision

The precision of the nanofibre size is critical to assess, considering that uniformity of size is important to ensure the characteristic of fuel cell performance that mass transfer analysis is done through a certain surface area or diameter. Less precision value means that the fibre structure has a higher uniformity than more precision value with inconsistent structures. The precision in this work was calculated by the standard deviation of diameter.

Based on Tables 2, it could be seen that the most uniform fibre was produced from experiment no. 6 (*S*: 1000, *V*: 10, *D*: 12, *M*: 20). Meanwhile, experiment no. 2, produce the most irregular fibre dimensions (*S*: 800, *V*: 15, *D*: 10, *M*: 20). Based on the S/N ratio (Fig. 4(D)), it can be concluded that the most precision nanofiber will be achieved at *S*: 1200, *V*: 10, *D*: 8, *M*: 0.

Based on the S/N result, the voltage becomes the most influential factor in achieving uniform fiber dimension. It could be seen from the S/N ratio that the precision of fibres fabricated by electrospinning is higher when the voltage decreases. The reason behind this phenomenon is the electrospinning process, during which the polymer solution is subjected to a high electric field, which causes the solution to be ejected from the spinneret in the form of a jet.^44^ At lower voltages, the jet instability is reduced, leading to more stable and consistent fibre formation. At lower voltages, the reduced electric field intensity results in less dramatic whipping and stretching of the jet, allowing for more controlled fibre formation and higher precision.

### 3.2. Effect of set-up parameters and their analysis

In this system, the voltage played a dominant role in all responses (DoA, diameter, bead, and precision). It should be noted that other factors also play a role in this fabrication process. The concentration that leads to viscosity, surface tension, or other fluid characteristics might change further. For instance, the viscosity should increase if the nanofiber is targeted at a larger size. This condition could be achieved by increasing the concentration of PVA or changing PVA with a higher molecular weight. To enlarge the understanding of how parameters involve the nanofiber structures, the discussion regarding rotation, voltage, distance, and exposure is provided in this section.

#### 3.2.1. Speed of rotation

Rotation is the advanced setup parameter of electrospinning, considering that the collector typically implements flat metal. It has been reported that increasing the speed of the collector increases the degree of alignment. In this work, the increasing rotation speed also increased the orientation of nanofiber (see Fig. 4). It has been reported that the degree of alignment in the electrospinning process is affected by the speed of the collector.^45–47^ In this work, the result found that the higher the speed, the higher the degree of alignment. This result strengthens the previous research that increasing the speed of rotation could increase the orientation of the nanofiber. It has been reported that increasing the speed of the collector boosts the fibre alignment by employing larger shear forces on the polymer jet. This report also revealed that rotation also decreases the fibre diameter by stretching the polymer jet.^48^ In this work, it has been recognized as the factor that can align the nanofibers. In this research, the higher the speed, the higher the degree of alignment.

**Fig. 6:**
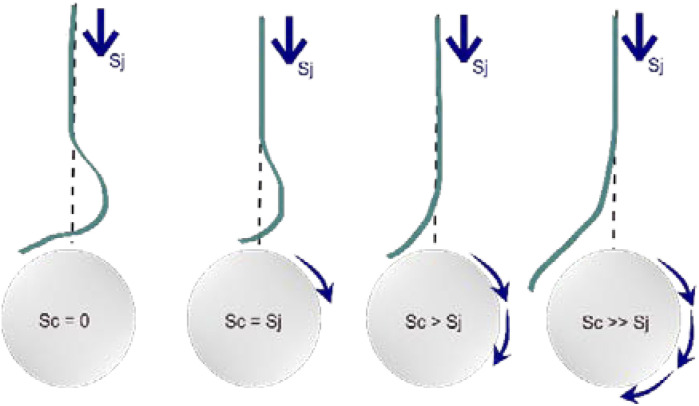
Relationship between the relative speed of the jet and the speed of the collector.^50^

Fig. 6 illustrates how the rotation influences the structure of the nanofiber. This process implements high shear forces experienced by the jet during collection, which can orient the polymer chains. In this work, the collector speed is perpendicular to the jet speed. Therefore, the jet profile could be assumed as a vertical deposition to a rotating collector. This event has been elaborated, revealing the relationship between jet speed (SJ) and collector speed (SC).^49^ It can be seen in Fig. 6 that the change in the shape of the jet profile corresponds to four different relative collector speeds. When the collector has a stationary (*S*_C_ = 0) speed, the jet's path is straight with a compressive heel close to the collector where buckling occurs. This condition causes the location of deposition to diverge away from the point directly below the spinneret as coiling occurs. When the collector speed increases, the net effect of the compressive force is reduced. Under the condition that the speed is equal to the jet speed (*S*_C_ = *S*_J_), the heel region in the jet profile has decreased, balancing the tensile and compressive forces. If we increase the speed above the jet speed (*S*_C_ > *S*_J_), the point of contact between the jet and the collector begins to “lag” behind. This condition happens because the dragging movement overcomes the compressive stresses, affording a delayed response of the stretched polymer. When the speed of the collector is way faster than the jet speed (*S*_C_ ≫ *S*_J_), further stretching of the jet occurs.^50^

It has been seen that when the collector rotates at a higher speed, the polymer jet must cover a more considerable distance at the same time. This phenomenon leads the jet to greater elongation forces, making the fibre stretch and thinner before it reaches the surface of the collector. In principle, the higher collector speed “pulls” the jet more stretched, resulting in a decreased final fibre diameter.

#### 3.2.2. Applied voltage

**Fig. 7:**
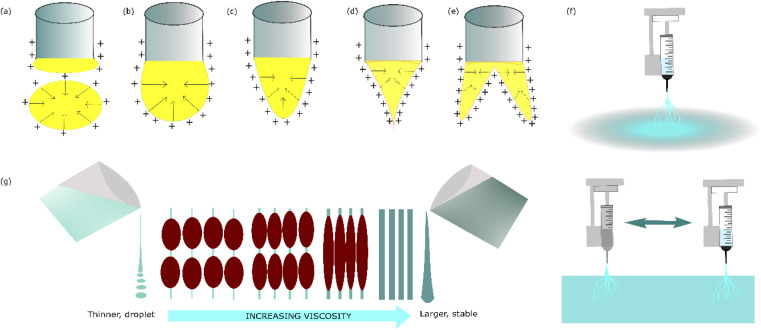
Taylor cone profile related to ion charge during electrospinning fabrication: (a) droplet, (b) bulb, (c) isobaric, (d) tapering, and (e) splinter. (f) Correlation between viscosity, diameter and bead formations.^60^ (g) Differences between a permanent nozzle and moving nozzle for fabrication.

In this work, the voltage play is dominant in all the responses. Without concentration factors, these parameters become superior to each other. Remembering that electrospinning works by implementing the electrostatic force. The voltage becomes the source to stretch and elongate the liquid to become a fibre. The effect of the ion charge related to the cone profile is illustrated in Fig. 7. It has been reported that increasing the voltage enhances the electrostatic force on the polymer jet. This setup leads to higher alignment. In this work, voltage play is dominant in all of the responses. The voltage becomes the source to stretch and elongate the liquid to become a fibre. Different applied voltages generate different ion charges that shape different profiles of the Taylor cone. The effect of the ion charge related to the cone profile is illustrated in Fig. 9(a).

Electrospinning uses generating voltage to create electrostatic force in the electrospinning process. When the voltage increases in the system, a higher electric field occurs, pulling force on the polymer moving the liquid from the tip to the collector. This pulling force decreases stretching, making polymer deposits thicker and larger nanofibers.

The relationship between the Taylor cone formation and the voltage occurs when there is a potential difference between the spinneret and the collector applied. This potential difference will cause the liquid to migrate toward the surface of the droplet and make excess charges. When the voltage is increased, more charges will accumulate, affecting the density of surface charges and promoting the droplet. In this event, the surface tension tends to form a spherical shape, minimizing the total surface free energy of the droplet. Crucially, the electrospinning process depends on the critical voltage needed to form a conical shape. When a liquid polymer is applied to the system, the magnitude of the voltage must reach a critical threshold to generate an electrostatic repulsion that is strong enough to surpass the surface tension and the viscoelastic properties of the liquid. This conical shape can be maintained by providing a sufficient flow rate of liquid that is ejected during an electrospinning process.^51^

#### 3.2.3. Distance between the tip and the collector

Proper distance in the electrospinning process is not just a matter of procedure, but it is also a matter of outcome. The liquid must evaporate, and this is directly influenced by the distance. A shorter distance strengthens electrical forces, while a longer distance prolongs evaporation, leading to a thinner diameter. Moreover, a longer distance can decrease the effect of the electrostatic force that causes droplets, potentially impacting the quality and properties of the final product.

Another parameter, a more significant distance between the tip and the collector, can also increase the degree of alignment.^52^ The more considerable distance leads to a longer time, allowing for better jet stretching and making orientation structure. Proper distance in the electrospinning process is not just a matter of procedure, it's a matter of outcome. The liquid must evaporate, and this is directly influenced by the distance. A shorter distance strengthens electrical forces, while a longer distance prolongs evaporation, leading to a thinner diameter. Moreover, a longer distance can decrease the effect of the electrostatic force that causes droplets, potentially impacting the quality and properties of the final product. It has been reported that a longer distance between the tip and the collector permits more time for the polymer jet to stretch and elongate, resulting in thinner fibre diameters.^53^ In this work, the distance has become an essential factor due to weakening or strengthening of electrostatic force. In this work, a higher distance tends to produce a higher aligned nanofiber. The longer distance provides more space for the polymer to experience bending instability.^51^ As the fibre moves over a longer distance, the whipping motion and stretching polymer become more maintained.

Regarding the distance, in the nanofiber fabrication process, the polymer experiences elongation and stretching of the polymer solution which has strong correlation with the diameter. In this elongation process, the nanofibers are exposed to an elongation force to reduce the size of the fibres.^54^ When nanofibers are close together, the surrounding fibres can interfere with this elongation process, causing the fibre diameter to become thicker. In this elongation process, the evaporation process causes the fibre to become thinner.^55^ At close range, the nanofibers have time to make the fibres thinner. However, at close range, the nanofiber has thicker dimensions.

It has been generally acknowledged that the diameter of the polymer jet in a straight line decreases with the distance away from the tip when it is continuously stretched. It has been mentioned that when the acceleration drops to zero or a constant, any small perturbation can destroy the straight movement, which has consequences producing instability.^56^ It has been reported that different types of instabilities could occur corresponding to the electrically charged jet, including Rayleigh (axisymmetric) instability, which may lead to the breakup of the jet into droplets and whipping or bending instability, which are non-axisymmetric results from the electrostatic repulsion among surface charges in a strong electric field.^57^

It has been analyzed that the fabrication of ultrathin nanofibers by electrospinning can be achieved using the rapid growth of whipping instability, so that this process can bend or stretch the jet.^58^ Under this condition, most of the elongation occurs in the loop due to the bending movement. The polymer jet will bend and elongate, and on further elongation, the elements in the jet elongate into thinner segments, resulting in much smaller coils and triggering the formation of another bending instability stage. If the jet solidifies before the second bending instability occurs, the loop diameter during the first bending instability no longer increases. As a result, the envelope cone becomes an “envelope cylinder”. In another phenomenon, constant solvent evaporation can reduce the volume and change the viscoelastic parameters of the jet in its path, making it difficult to stretch the jet further, so that the loop diameter will become smaller.^51^

#### 3.2.4. Movement nozzle

Movement is the parameter regarding the movement of nozzle of the electrospinning system. This response is essential to be investigated, considering that this factor could influence the structure of nanofibers. When electrospinning only makes one deposition, the thickness is concentrated at the centre of the collector. In this work, Tong Li Tech is the apparatus that could provide the nozzle movement to give the uniformity of thickness, so that, this parameter could be added as a new factor to be investigated.

Based on data, nozzle movement has effect in increasing the degree of alignment, increasing the fibre diameter variation, reducing the bead generation, and has strong relation to the precision of the fibre size.

The nozzle movement is an essential factor in nanofiber outcomes because this approach makes the thickness of the nanofiber in the collector uniform. When the length of exposure is not implemented, the thickness of the fibre is concentrated at the centre of the collector, leading to a difference in thickness that is an undesired property in MEA fabrication. Fig. 7(f) illustrates the differences between the permanent nozzle and the movement nozzle.

### 3.3. Correlation between parameters and structures

In this experiment, the fluid parameters settled in constant value because the concentration is settled as a permanent value. The interaction of four factors in this experiment is essential to understand the phenomenon behind the result. It should be noted that, in this work, the voltage comes as the most significant factor. The electrostatic force generated by high voltage supply needs to be broken down.

It has been reported that electrostatic force formula mentioned the attribute of its process. The force in the system can be formulated as eqn (4):^59^

4 *F*_C_ − *F*_γ_ + *F*_H_ = 0

*F*_C_ is the coulombic force that occurs because of the electric field. *F*_γ_ is the force caused by surface tension that appears from the charge in the polymer. Meanwhile, *F*_H_ is the hydrodynamic force that occurs because of the pressure on the polymer.

Based on the formulation, when the total force is zero, a half spherical surface would appear in the hole of syringe. Then, it is changed to cone shape because it is pulled by the electric field. In a certain distance, this phenomenon made polymer jet to the ground. In the short distance, the jet is a straight line because of jet effect. However, in the next event, the fibre becomes thinner and smoother caused by the evaporation of the solute polymer. In this line, interaction between the fibre, that is charged, and the air occurred extensively causing non-woven polymer fibres.

During jet movement, along the *z*-axis, the continuity of mass occurs, meanwhile the mass velocity is constant. It can be formulated as eqn (5):^59^5
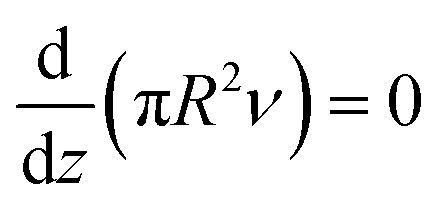
*R* represents the tip diameter and *ν* is the velocity of jet. Through jet movement, the current electricity is also constant, and it could be represented as eqn (6):^59^6
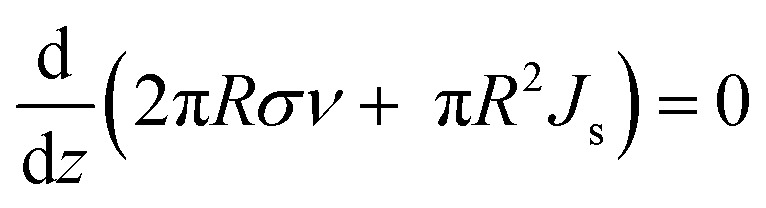
where *σ* is the area charge density and *J*_s_ is the spherical area current density. Furthermore, through jet movement also occur the law of conservation of momentum, represented as eqn (7):^59^7
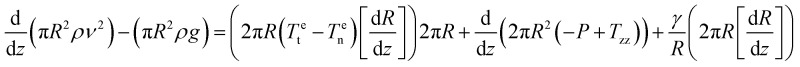


The first term on the left side represents the voltage effect electric surface on the tangential surface *T*^e^_t_ and normal *T*^e^_n_, whereas the second term is the voltage surface of jet. The electric field during the jet process can be formulated as eqn (8):^59^8
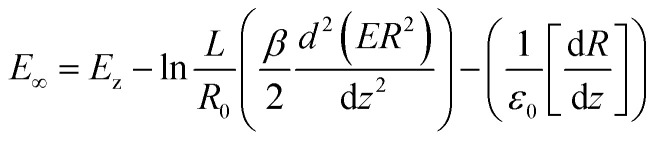
where *E*_∞_ is the external field and *E*_z_ is the electric field along the *z* axis. In this system, the viscosity of the polymer used affects the fibre yield because during a jet occurs, there is a change in polymer stress in the radial direction and axial directions that couple each other so that the speed the jet direction in the *z*-axis is different from the speed in the radial direction. This effect is expressed through eqn (9):^59^
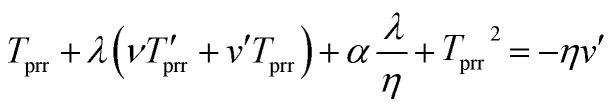
9
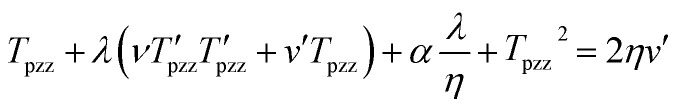
where 
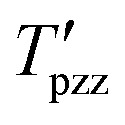
 represents the stress on the *z* axis and 
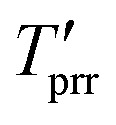
 is the stress on the radial axis, *λ* is the relaxation time and *α* is the polymer mobility. Eqn (5)–(9) with appropriate boundary conditions can be used to calculate the size of the polymer radius when the jet occurs. However, in practice after a jet occurs, there will be evaporation of water from the polymer and interactions between the polymers, so that the fibre is not straight. The size of the fibre will be the smaller, linear to the distance between the syringe's tip and the collector.

Regarding bead generation, this structure appears in nanofibers due to some factors, for instance, the surface tension between the air and the polymer. It has been mentioned that bead generation is closely related to diameter. When the bead formation appears, the diameter fiber close to the bead tends to be thinner. This feature also indicates that the condition of process fabrication nanofiber is at borderline between the fibre and the droplet. Bead formation in electrospinning is typically associated with low solution viscosity. Fig. 7(g) illustrates the relationship between the viscosity, diameter, and bead formation.^60^ Applied voltage and jet movement can have a significant impact on bead generation. Higher voltages can increase bead formation, as the greater electric field strength can cause jet instabilities.^48^ In this work, experiment 6 reveals that if the distance is longer and the voltage is lower, the droplets appear because the inlet of the flow rate is higher than the stretched process. The distance has become an important factor due to weakening or strengthening of the electrostatic force. In this work, experiment 6 reveals that if the distance is longer and the voltage is lower, the droplet appeared because the inlet of the flow rate is a higher stretched process.

The significance rank of factors was conducted to particularise the effect of set-up parameters on actual response using ANOVA regression analysis conducted using the Minitab Software. Table 3 reveals the level of effect on each response. In this system, the voltage played a dominant role in all responses (DoA, diameter, bead, and precision). It should be noted that other factors also play a role in this fabrication process. The concentration that leads to viscosity, surface tension, or other fluid characteristics might change further. For instance, the viscosity should increase if the nanofiber is targeted at a larger size. This condition could be achieved by increasing the concentration of PVA or changing PVA with a higher molecular weight.

**Table 3:** Correlation between factors, responses, and their significance quantified using ANOVA

Factors	DoA		Diameter		Bead		Precision	
	Rank	Signf. (%)	Rank	Signf. (%)	Rank	Signf. (%)	Rank	Signf. (%)
Speed	4	4.17	3	17.64	3	1.39	2	25.53
Voltage	1	29.88	1	38.2	1	63.86	1	42.04
Distance	2	23.33	4	4.3	4	0.034	3	0.06
Movement	3	12.33	2	18.92	2	16.11	4	0.16
Others	>1	30.29	>2	20.9	>2	18.61	>2	30.2

**Fig. 8:**
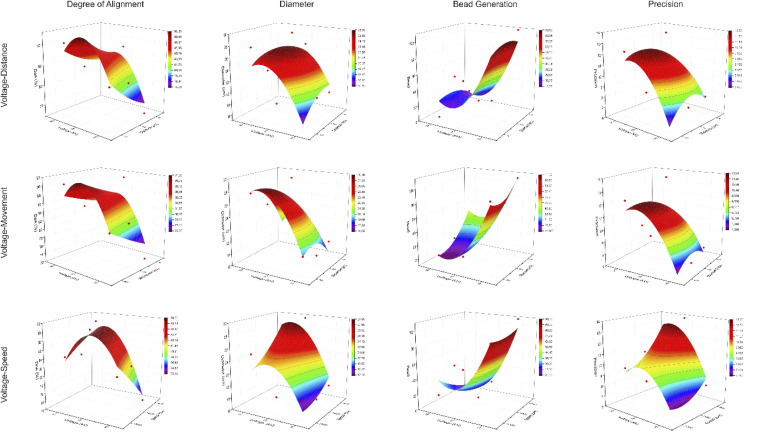
Heat map surface as a response of the nanofiber structure correlated with significance factors.

To analyze the relationship between factors and responses, a heat map surface is provided to see the profile of the electrospinning system, especially to see the behavior of parameters in the thinner size (around 20 nm), which is mainly affected by voltage. Fig. 8 presents the heat map surface of the four responses corresponding with the two most significant factors. Each response's different surface indicates that these parameters orchestrate the electrospinning process. The colder color indicates a lower value, while the hotter color indicates a higher response value.

### 3.4. Optimization structure: selecting and validating result

In this work, there is a reversed trend between degree of alignment, diameter precision *versus* bead diameter. Meanwhile, the goal of these results is to investigate the structures with the finest characteristics based on the degree of alignment, diameter, bead formation, and precision.

The first parameter, namely speed, shows that two maximum values appear in thin and less bead generation. Meanwhile, the speed is surprisingly optimum for the lowest rpm setup. It should be noted that Taguchi's design is not a full factorial experiment; we must be concerned that some specific parameters are significant to another. Based on Table 3, the speed in the case of alignment is less significant. For optimum conditions, the speed will be set at a maximum value of 1200 rpm. The second parameter, voltage, also makes problematic decisions. For more oriented alignment and less diameter, the maximum value should be chosen. However, to generate fewer beads and produce more precision, the lowest value should be chosen. Bead generation and precision are closely related to fluid parameters. Increasing the concentration to increase the viscosity of the system could deal with the problem. Therefore, the highest value of the voltage is chosen to be optimized with the concern of changes at higher concentrations. Regarding the distance, the highest distance, 12 cm, appears to influence alignment and less bead formation, even though a lower value, 8 cm, appears in the precision output. Based on the significant value, that is only 0.06%. This consideration can be neglected. Regarding movement, the lowest value has a role in bead formation, and the highest value appears in DoA, diameter, and precision. However, the movement has a crucial role in the uniformity of thickness, and the movement of the nozzle does not have any crucial role in determining the damage to the product.

The tread off must be ensued to find the desired nanofiber. Nonetheless, by compiling the S/N ratio in Fig. 4 and the correlation between significant factors in Fig. 8, the recommendation towards the finest structure is a higher speed of rotation collector, properly applied voltage, longer distance between the nozzle and the collector, and wider nozzle movement. The reference parameter leads to a more oriented, smaller, less bead and uniform nanofiber structure. Considering the SEM result in Fig. 3, images no 1, 4, and 7 represent the highest value of applied voltage, and images 3, 6, and 9 are considered as the lowest value of applied voltage; it is witnessed that a higher applied value gives a better result than a lower value.

**Fig. 9:**
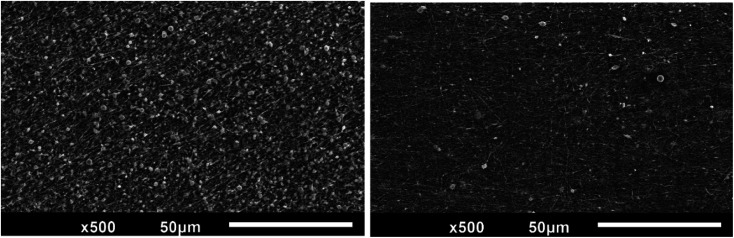
Trade off between degree alignment and bead formation in the electrospinning system.

**Fig. 10:**
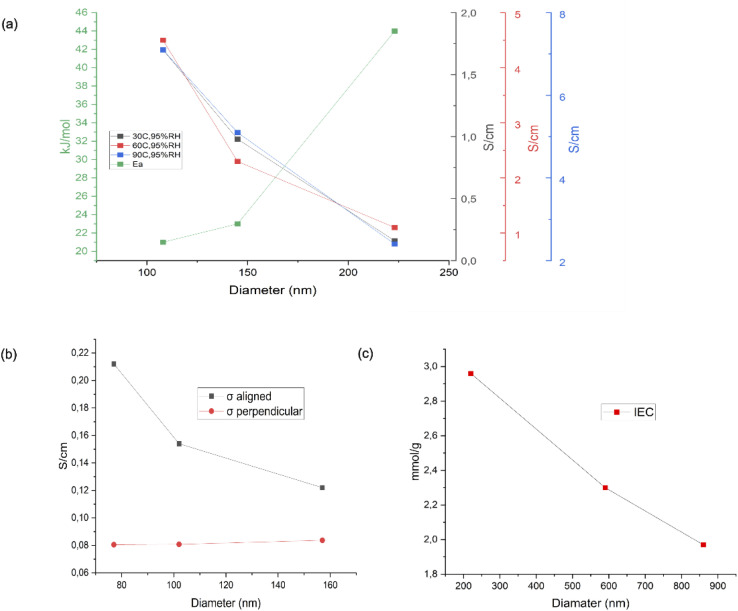
The reported result of the effect of various diameters of nanofibre on characteristic parameters of PEMFCs, including (a) proton conductivity and activation energy,^69^ (b) proton conductivity,^70^ and (c) IEC.^71^

The thread off between the alignment and bead formation can be seen in Fig. 10. When the alignment is higher, the bead generation is also increased. Meanwhile, when the bead generation is less, the degree of alignment is reduced. In Fig. 9 (left), it can be observed that the degree of alignment is presented nicely. However, the bead generation is also highly presented. This condition occurs when the speed of rotation and the distance have the highest value. Compared to Fig. 9 (right), it can be seen that the bead is less generated but the alignment is poor. This occurs when the voltage is set to a higher value.

### 3.5. Potential outlook: towards MEA fuel cells

Regarding the material used in this experiment, PVA has been widely implemented as a membrane for developing anion exchange membranes.^29,61,62^ This polymer has advantages in hydrophilicity properties, providing high water uptake and a high density of reactive chemical groups.^63^ PVA-based membranes are treated with additives in the preparation stage to enhance membrane superiority, including a cross-linker for restraining membrane stability and improving mechanical strength,^64^ an inorganic filler to enhance thermal and electrochemical properties,^65^ a plasticizer for decreasing stiffness,^66^ and an ionic liquid to increase the ionic conductivity.^67,68^

The comparison of different diameters in regard to fuel cell characteristics has been reported. Fig. 10(a) shows the relationship between various diameters and the fuel cell performance and characteristics. It is described that the thinner nanofiber has a higher proton conductivity and a lower activation energy.^69^Fig. 10(b) also presents the relationship between the nanofiber diameter and its orientation related to proton conductivity. The results strengthen that the thinner nanofiber which has aligned orientation has better proton conductivity.^70^Fig. 10(c) explains the relationship between various nanofiber dimensions and Ion Exchange Capacity (IEC). It has been shown that the thinner fiber has a higher IEC.^71^

In MEA applications, such as fuel cells, the nanofiber diameter can have consequences on the specific surface area and porosity. Smaller nanofibers can provide higher surface area-to-volume ratios, enabling more efficient ion diffusion. For the catalytic activity, this surface area and accessibility of the nanofibers are critical factors. By increasing the specific surface area, the catalytic activity can be improved, allowing for more efficient ion adsorption/desorption and redox reactions.^72,73^ Through careful control of the nanofiber diameter and manipulation of parameters, such as tuning the interfibrous spacing, the pore size distribution can be tailored to optimize the balance between ion diffusion kinetics. Larger pore sizes facilitated by greater nanofiber distances can enhance ion transport. Nanofiber-based electrodes, with their inherent flexibility and resilience, can offer improved mechanical stability and flexibility. Adjusting the nanofiber distance and diameter can help optimize the mechanical properties of the electrode, ensuring its integrity and long-term performance.

Aligning the nanofiber diameter with the MEA structure can help optimize the fuel cell's performance. The alignment nanofiber also helps to minimize mass transport limitations and improve the overall utilization of the catalyst.^74,75^ Proper alignment of nanofiber diameter with the MEA structure can lead performance improvements. The robust nanofiber structure can help maintain the integrity of the electrode and the MEA, leading to better long-term stability and durability. The nanofiber structure can facilitate effective water management.^76^ Within the fuel cell application, this property prevents flooding or drying out of the membrane.

The diameter of nanofibers used in the electrodes of a fuel cell MEA can have a significant impact on the overall performance and efficiency of the fuel cell. It has been reported that smaller diameter nanofibers are preferred for fuel cell MEAs. Smaller nanofibers offer a larger surface area-to-volume ratio, which can enhance the catalytic activity and improve the oxygen reduction reaction (ORR) kinetics at the cathode. The nanofiber diameter should be small enough to penetrate the catalyst layer and the membrane, ensuring good contact between the electrode and the electrolyte. Smaller nanofibers also create a more efficient network for electron and proton transport within the electrode structure. In this work the fibre is smaller than the latest development. The finding of this work could lead to potential improvement in fuel cell performance development.

## 4. Conclusions

Summarizing the findings of this study, voltage is found to be the most significant factor in fabricating ultrathin nanofibers in all assessed profiles including the degree of alignment, diameter, bead generation, and precision.

Optimizing the electrospinning process and tailoring the fibre properties to meet the desired application are required for adjusting the parameter setup, and the effects are as follows:

(1) Effect of the rotational speed of the collector: increased rotational speed leads to higher shear forces, resulting in greater fibre alignment and smaller fibre diameter, as the shear forces align the polymer chains and reduce the overall fibre diameter.

(2) The applied electrical voltage: a higher voltage generates a higher electrostatic force which exerts a pulling force on the polymer to move the liquid from the tip to the collector. This pulling force decreases stretching, making polymer deposits thicker and nanofibers larger. However, the excessively high voltage also causes jet instability and bead formation.

(3) The distance between the nozzle and the collector affects the time for jet elongation, and solvent evaporation leads to smaller fibre diameters. Too large a distance creates jet instability and incomplete solvent evaporation.

(4) The movement of nozzle strongly influences the uniformity of the size of nanofiber, and this parameter is also essential to produce uniform thickness of nanofibers.

In this study, the diameter of the nanofiber was found to be thinner than the current MEA application using nanofibers. Compared to the existing MEA applications that have a size around 30–500 nm,^38–40^ this work successfully fabricates nanofibers of size around 20 nm on average. This discovery is significant as thinner nanofibers can lead to a higher surface area and porosity. The findings of this work reveal that the nanofibers hold great potential to enhance the performance of MEAs in fuel cells.

## Data availability

The authors confirm that the data supporting the findings of this study are available within the article.

## Author contributions

Conceptualization, M. Y.; investigation, M. Y.; writing—original draft, M. Y.; writing—review and editing, M. Y. and V. H.; supervision, V. H.; funding acquisition, V. H. all authors have read and agreed to the published version of the manuscript.

## Conflicts of interest

There are no conflicts to declare.
